# A novel coumarin derivative DBH2 inhibits proliferation and induces apoptosis of chronic myeloid leukemia cells

**DOI:** 10.1016/j.gendis.2022.08.021

**Published:** 2022-09-08

**Authors:** Jiajia Xin, Huijie Zhang, Dandan Yin, Ning An, Yaozhen Chen, Jinmei Xu, Jing Zhang, Zhixin Liu, Yongsheng Liu, Wen Yin, Mingkai Li, Xingbin Hu

**Affiliations:** a Department of Blood Transfusion, Xijing Hospital, Air Force Medical University, Xi’an, Shaanxi 710032, China; b Key Laboratory of Biochemistry and Molecular Pharmacology of Chongqing, Chongqing Medical University, Chongqing 400016, China; c Department of Hematology, Tangdu Hospital, Air Force Medical University, Xi’an, Shaanxi 710038, China; d Department of Pharmacology, School of Pharmacy, Air Force Medical University, Xi’an, Shaanxi 710032, China

**Keywords:** Apoptosis, Caspase, Chronic myeloid leukemia, Coumarin, STAT

## Abstract

With the development of tyrosine kinase inhibitor (TKI) resistance, finding the novel effective chemotherapeutic agent is of seminal importance for chronic myelogenous leukemia (CML) treatment. This study aims to find the effective anti-leukemic candidates and investigate the possible underlying mechanism. We synthesized the novel coumarin derivatives and evaluated their anti-leukemic activity. Cell viability assay revealed that compound DBH2 exhibited the potent inhibitory activity on the proliferation of CML K562 cells and TKI resistant K562 cells. Morphological observation and flow cytometry confirmed that DBH2 could selectively induce cell apoptosis and cell cycle arrest at G2/M phase of the K562 cells, which was further confirmed on the bone marrow cells from CML transgenic model mice and CD34^+^ bone marrow leukemic cells from CML patients. Treatments of DBH2 in combination with imatinib could prolong the survival rate of SCL-tTA-BCR/ABL transgenic model mice significantly. Quantitative RT-PCR revealed that DBH2 inhibited the expression of STAT3 and STAT5 in K562 cells, and *caspase-3* knockout alleviated the DBH2 induced apoptosis. Furthermore, DBH2 could induce the expression of PARP1 and ROCK1 in K562 cells, which may play the important role in caspase-dependent apoptosis. Our results concluded that coumarin derivative DBH2 serves as a promising candidate for the CML treatment, especially in the combination with imatinib for the TKI resistant CML, and STAT/caspase-3 pathway was involved in the molecular mechanism of anti-leukemic activity of DBH2.

## Introduction

Chronic myelogenous leukemia (CML) is a hematopoietic stem cell disorder characterized by the Philadelphia chromosome, the result of a gene mutation of translocation between chromosomes 9 and 22, which fuses the BCR gene with the ABL1 gene and produces the constitutively active BCR-ABL1 (Bcr-Abl) tyrosine kinase, directly leading to the proliferation of leukemia cells.^1^ These groundbreaking scientific studies have led to the rapid development of many BCR-ABL specific tyrosine kinase inhibitors (TKIs) such as imatinib, which improved 10-year survival to more than 80%.^2^ Unfortunately, approximately 20%–30% of patients did not respond optimally to TKIs therapy, and alternatively spliced BCR-ABL1 mRNA with 35-nucleotide insertion was found in CML patients who showed resistance to imatinib.^3^ Additionally, loss of responses in about 5% CML patients was due to BCR-ABL-independent mechanisms.^4^

It was reported that overexpression of signal transducer and activator of transcription (STAT) plays an important role in resistance to apoptosis and carcinogenesis including the CML, and alternative STAT signaling pathway was involved the TKIs resistance in CML cells.^5^^,^^6^ Therefore, unraveling the detailed molecular mechanisms and finding the novel effective chemotherapeutic agents are of seminal importance for CML treatment after TKI failure.

Coumarin is a versatile synthetic scaffold possessing wide spectrum of biological activity, and increasing evidence showed that coumarin derivatives exerted activity against various carcinoma cell lines including breast cancer,^7^^,^^8^ lung cancer^9^ colon cancer^10^ and ovarian cancer.^11^ Hence, the coumarin derivatives have the promising potential to be the anti-cancer agent. However, less is known about the effect and possible mechanism of coumarin derivatives in the CML. In recent years, research showed that inhibition of erythroblastic leukemia viral oncogene homolog 2 (ErbB-2) phosphorylation was involved the anti-tumor mechanism of coumarins, which was correlated with down-regulation of regulated protein kinase (ERK) activation in breast cancer cells.^12^ While suppressing ROS-independent c-Jun N-terminal protein kinase (JNK), but not extracellular ERK or p38, substantially diminished coumarins-induced cell death in human colon cancer cells.^13^ Interestingly, it was also reported that some coumarins had the activities of antioxidant and inhibition of ROS-producing enzymes.^14^ However, the molecular mechanism of coumarin-induced apoptosis was not fully understood.

In the present study, we synthesized six novel coumarin derivatives, and measured the inhibitory activity of these compounds on the proliferation of CML K562 cells and TKI resistant K562 cells. Then we studied the possible role of the most potent derivative (compound DBH2) on the apoptosis of CML cells, bone marrow cells from BCR-ABL transgenic mouse and human patients. Furthermore, we observed the survival of BCR-ABL transgenic animal models after the treatment of DBH2 combined with imatinib, and explored the possible role of STAT, caspase-3 and downstream signaling molecules. These studies provided the potential therapeutic candidate for CML, especially for those patients who developed into TKI resistant, and revealed the possible underlying molecular mechanism of coumarin derivative through caspase-3 mediated apoptosis.

## Methods and materials

### Synthesis and characterization of coumarin derivatives

Coumarin derivatives were synthesized according to the methods of our previous report.^15^ Briefly, a mixture of 10 mmol 3-methoxybenzaldehyde and 20 mmol 4-hydroxycoumarin was dissolved in 100 mL ethanol. A few drops of piperidine were added, and the mixture was stirred for 3 h at room temperature. After the completion of reaction, water was added until precipitation occurred. After filtering the precipitates, they were sequentially washed with ice-cooled water, ethanol and dried in a vacuum. IR spectra (400–4000 cm^−1^) were obtained using a Brucker Equinox-55 spectrophotometer. ^1^H NMR spectra were obtained using a Varian Inova-400 spectrometer (at 400 MHz). Mass spectra were obtained using a micrOTOF-Q II mass spectrometer. The melting points were taken on a XT-4 micro melting apparatus, and the thermometer was uncorrected. The compounds were listed as below, the synthetic route and the chemical structures were shown in Figure S1.

**Compound DBH1: 3,3′-benzylidene-bis-(4-hydroxycoumarin)**^1^H NMR (CDCl_3_, *δ*, ppm): 11.528(s, 1H), 11.299(s, 1H), 7.994–8.080(q, 2H), 7.606–7.649(m, 2H), 7.215–7.421(m, 9H), 6.104 (s, 1H).

**Compound DBH2: 3,3′-(3, 5-difluorobenzylidene)-bis-(4-hydroxycoumarin)**

^1^H NMR (CDCl_3_, *δ*, ppm): 11.666(s, 1H), 11.323(s, 1H), 8.033–8.109(q, 2H), 7.660–7.701(m, 2H), 7.427–7.462(t, 4H), 6.731–6.790(m, 3H), 6.051(s, 1H).

**Compound DBH3: 3,3′-(3,5-dichlorobenzylidene)-bis-(4-hydroxycoumarin)**

^1^H NMR (CDCl_3_, *δ*, ppm): 11.630(s, 1H), 11.314(s, 1H), 8.042–8.114(m, 2H), 7.666–7.705 (q, 2H), 7.432–7.468(t, 4H), 7.307–7.309(d, 1H), 7.106–7.113(t, 2H), 6.036(s, 1H).

**Compound DBH4: 3,3′-(3, 5-dibromobenzylidene)-bis-(4-hydroxycoumarin)**

^1^H NMR (CDCl_3_, *δ*, ppm): 11.616(s, 1H), 11.306(s, 1H), 8.042–8.111(q, 2H), 7.666–7.705(t, 2H), 7.608(s, 1H), 7.411–7.467(q, 4H), 7.294(s, 2H), 6.049(s, 1H).

**Compound DBH5: 3,3′-(3, 5-dimethoxybenzylidene)-bis-(4-hydroxycoumarin)**

^1^H NMR (CDCl_3_, *δ*, ppm): 11.645(s, 1H), 11.269(s, 1H), 8.027–8.090(m, 2H), 7.628–7.671(m, 2H), 7.417–7.438(d, 4H), 6.393(s, 3H), 6.060(s, 1H), 3.743(s, 6H).

**Compound DBH6: 3,3′-(3, 5-ditrifluoromethylbenzylidene)-bis-(4-hydroxycoum -arin)**

^1^H NMR (CDCl_3_, *δ*, ppm): 11.527(s, 1H), 11.403(s, 1H), 7.996–8.101(q, 2H), 7.814(s, 1H), 7.644–7.695(t, 4H), 7.390–7.447(q, 4H), 6.132(s, 1H).

### Cell culture

Human CML cell line K562 cells and human umbilical vein endothelial cells (HUVECs) were purchased from ATCC (Manassas, USA). Drug resistant K562 (K562R) cells were provided by the Chinese Academy of Medical Science Hematology Institute. The cells were cultured in RPMI-1640 medium containing 10% fetal bovine serum (FBS) in cell culture flasks under a humidified 5% CO_2_ and 95% air atmosphere at 37 °C. Bone marrow (BM) cells from mice or patients were cultured in DMEM/F12 medium supplemented with 20% FBS.

### Purification of CD34^+^ hematopoietic stem and progenitor cells (HSPCs)

Human CD34^+^ HSCPs were obtained from the specimens of consenting healthy donors or CML patients at Xijing Hospital, and purified using Human Whole Blood CD34 Positive Selection Kit II (StemCell Technologies, China). Briefly, the human bone marrow was homogenized, and the top layering with Ficoll-Paque (GE Healthcare, USA) interphase containing immune cells and CD34^+^ HSPCs was collected and washed with PBS, then CD34^+^ HSPCs were purified according to the protocol of EasySep human CD34 positive selection kit (StemCell Technologies, China). CD34^+^ cells were cultured in StemSpan-ACF medium II supplemented with 50 ng/mL human stem cell factor, 10 ng/mL thrombopoietin, 20 ng/mL FMS-related tyrosine kinase 3 ligand, 20 ng/mL interleukin-6 and 1% penicillin-streptomycin solution.

### Basic information of CML patients

The ages of three male CML patients were 23, 45 and 49, all of them were newly diagnosed chronic phase, and before starting TKI therapy. Informed consent was obtained from all participants. This study adhered to the World Medical Association’s Declaration of Helsinki and its amendments for Ethical Human Research including confidentiality, privacy, and data management.

### Cell viability assay

Cell viability was examined using the cell counting kit-8 (CCK-8) assay (YEASEN China). In brief, cells (1 × 10^4^ cells/well) in confluent 96-well cell culture plates were treated with different concentrations of coumarin derivative (1, 5, 10, 25, 50, 100, 200, and 400 μg/mL) for 48 h. Then 10 μL CCK-8 reagents were added into every well for 2 h of incubation. Optical density (OD) was detected using the microplate reader (BioTek, Germany). The values were then fit into a non-linear regression curve and the IC_50_ was calculated using GraphPad Prism.

### Flow cytometry

Apoptotic cells were quantified with an FITC Annexin V Apoptosis detection kit (Becton Dickinson Biosciences, USA) according to manufacturer’s protocol. Briefly, cells at 1 × 10^5^ cells/mL were incubated with various concentrations (50, 100 and 200 μg/mL) of compound DBH2 for 24 h at 37 °C. Cells were harvested and re-suspended in the binding buffer, then stained with 5 μL Annexin V-FITC and 5 μL propidium iodide (PI) for 15 min at room temperature in the dark. The apoptotic index was immediately determined by FACSCalibur Flow Cytometer (Becton Dickinson Biosciences, CA). For the cell cycle, K562 cells were harvested and fixed in 75% ethanol overnight at 4 °C, then re-suspended in pre-cooling PBS containing 0.1 mg/mL RNase and 0.02 mg/mL PI for 30 min at 37 °C. After the treatment of compound DBH2 or 1% DMSO as vehicle control, the cell cycle was analyzed using the flow cytometry and Cell Quest software (BD Biosciences, USA).

### Transmission electron microscopy (TEM) analysis

CML cells were seeded and grown at 5 × 10^7^/mL in three flasks. After treated by compound DBH2 or 1% DMSO as vehicle control, cells were harvested and washed with 1 × PBS twice, and then added to 2.5% glutaraldehyde fixative for microtome sectioning using ultra-microtome (LKB, Sweden), and observed under the JEM-2000EX TEM (JEOL, China).

### SCL-tTA/BCR-ABL transgenic mice

According to the previously reported method,^16^ the transactivator protein tTA was placed under the control of the murine stem cell leukemia gene 3′ enhancer. Induction of BCR-ABL resulted in neutrophilia and leukocytosis, the BCR-ABL expression in stem and progenitor cells in SCL-tTA/BCR-ABL transgenic C57BL6 mice was induced by tet on/off polyI:C for 4 weeks, the peripheral hemogram and spleen index were examined after identifying genotypes in mice, and the distribution of hematopoietic lineage in bone marrow was detected by flow cytometry.

### CML-like model mice and *caspase-3* knock-out mice

The SCL-tTA-BCR/ABL transgenic model mice were used as donor, and sub-lethally irradiated (850 rad) wild type mice were use as receiver, once the bone marrow transplantation (BMT) was performed from the donor mice to the recipient mice, the CML-like model mice was established. After 8 weeks, the bone marrow reconstitution was confirmed, and the effect of treatments to the survival rates of recipient mice was observed. *Caspase-3* knock-out mice were kindly gifted from the lab of Professor Hua Han, the Fourth Military Medical University.

### Quantitative RT-PCR

RNA was extracted from cells using an RNA extraction kit according to the manufacturer protocol. After reverse transcription, the real-time PCR was performed for three independent experiments with a 7300 real-time PCR system (Applied Biosystems, USA) using SYBR Premix ExTaq (Takara, China). The relative expression of genes was calculated by the 2^-△△Ct^ method, with normalization to GAPDH levels. Amplified products were visualized with an Alpha Imager 2200 digital imaging system (Alpha Innotech, USA). The primers (forward and backward) used were depicted in Table S1.

### Western blot analysis

Cells were washed with Tris-buffered saline (TBS) and lysed by adding 100 μL of RIPA Lysis Buffer System containing protease inhibitors. Then, cell extraction was subjected to gel electrophoresis and transferred onto a polyvinylidene difluoride membrane (Millipore, Germany). After blocking with 3% BSA in TBS for 2 h, the membrane was probed with primary antibodies overnight at 4 °C, the antibody against PARP1 (Proteintech, USA) or ROCK1 proteins (Proteintech, USA) was diluted at 1:1000, and the antibody for GAPDH (Abcam, UK) was diluted at 1:1000. After secondary antibodies labeled with infrared dyes were added, the signals were visualized using the Odyssey Infrared Imaging System (Biosciences, USA).

### Statistical analysis

Statistical analysis was performed using SPSS 21.0. All data are presented as mean ± SEM, and differences were assessed using one-way analysis of variance followed by the Dunnett *t* test. The Student *t* test was used to compare between the two groups. Significant differences were accepted if the *P* value was <0.05.

## Results

### Coumarin derivatives inhibit proliferation of CML cells

To investigate the effect of coumarin derivatives to the proliferation of CML cells and the cytoxicity to the mammalian cells, the human CML cell line K562 cells, K562R cells, HUVECs and HSPCs were treated by six compounds at various concentrations ranging from 1 μg/mL to 400 μg/mL by CCK-8 staining method. The results showed that the coumarin derivatives could inhibit the growth of drug sensitive or resistant K562 cells, while compound DBH2 showed the most potent inhibitory activity on the proliferation of K562 cells and K562R cells with IC_50_ at 36.32 μg/mL and 38.79 μg/mL, respectively, while less cytotoxicity to the HUVEC_S_ and HSPCs were observed with IC_50_ at 232.15 μg/mL and 225.16 μg/mL, respectively. Although the K562 cells were very sensitive to imatinib with IC_50_ at 0.15 μg/mL, the value increased about 32 times on K562R cells. Doxorubicin is an anthracycline antitumor antibiotic used in a wide range of cancers including hematological malignancies. Although this chemotherapeutic inhibited the proliferation of K562 cells with lower IC_50_ (16.44 μg/mL), it had strong cytotoxicity to the HUVEC_S_ with IC_50_ at 8.76 μg/mL (Table 1). These results indicated that compound DBH2 could inhibit both imatinib sensitive CML cells and resistant CML cells *in vitro*, and had the much less cytoxicity on HUVECs or HSPCs comparing to the doxorubicin.

**Table 1:** Effect of coumarin derivatives on the proliferation of human cells.

Agent	IC_50_ (μg/mL)			
	K562 cells	K562 R cells	HUVECs	HSPCs
DBH1	31.28	33.42	85.66	76.35
DBH2	36.32	38.79	232.15	225.16
DBH3	36.48	45.71	52.32	72.42
DBH4	36.54	39.85	88.65	83.12
DBH5	30.45	34.36	50.29	46.38
DBH6	31.32	35.68	72.42	67.75
Imatinib	0.15	4.86	12.40	11.35
Doxorubicin	16.44	24.14	8.76	8.04

### Compound DBH2 induced the apoptosis of CML cells

**Figure 1:**
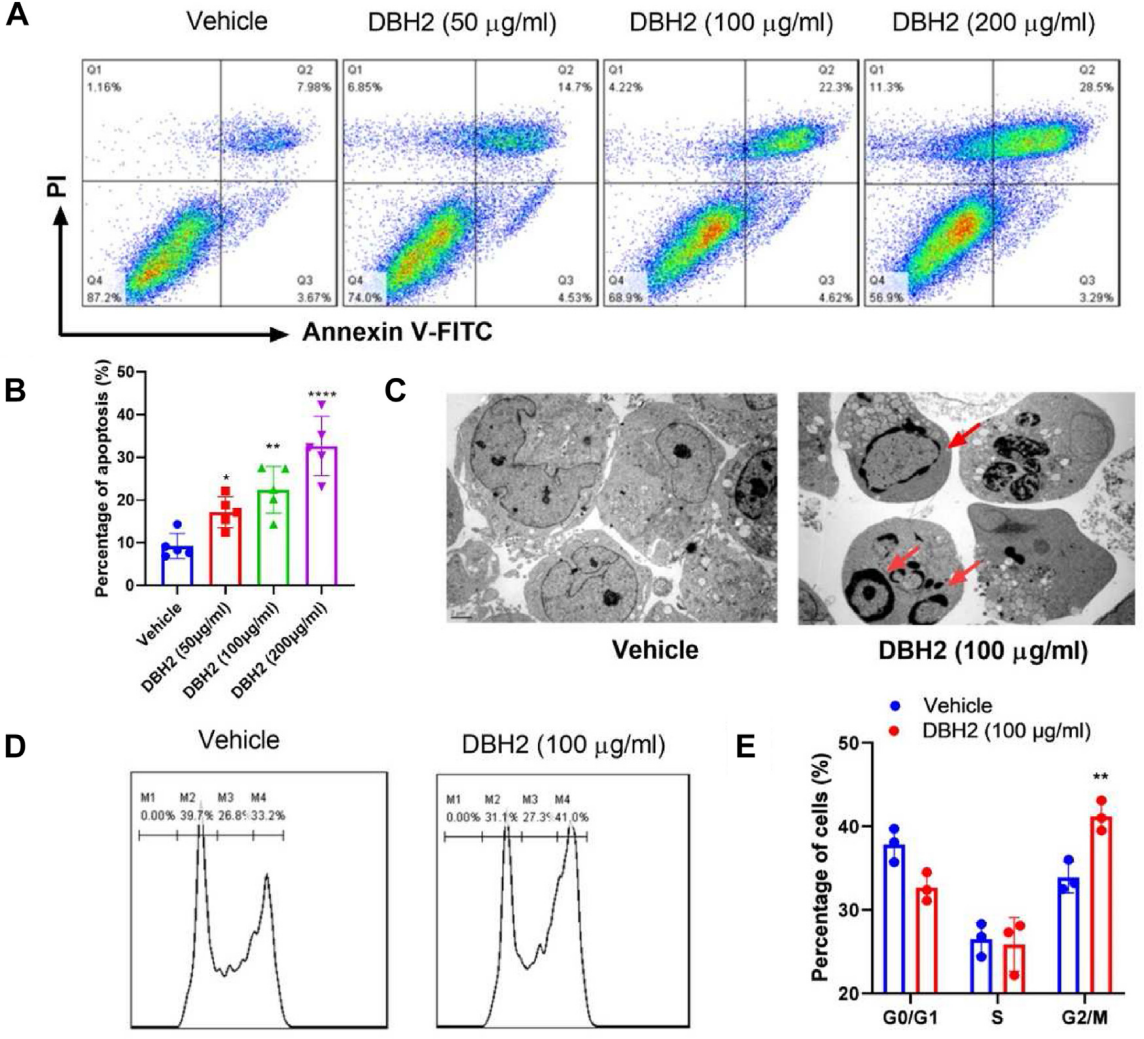
Compound DBH2 induced apoptosis of K562 cells and G2/M cell cycle arrest. **(A)** The representative flow cytometric results showed the apoptosis of cells by staining with FITC-labeled Annexin V and PI. **(B)** The percentages of apoptotic cells-based flow cytometric staining, ∗*P* < 0.05, ∗∗*P* < 0.01, ∗∗∗∗*P* < 0.0001 *vs*. vehicle control, *n* = 5. **(C)** Morphological observation was performed to show typical apoptotic characteristics in K562 cells after 100 μg/mL DBH2 treatment post 24 h under transmission electron microscopy (TEM). Arrows in red color indicated the apoptotic-body in nuclear, and magnification was 20,000 times. **(D)** The representative flow cytometry data of K562 cells after 100 μg/mL compound DBH2 treatment or 1% DMSO as vehicle control with Annexin-V and PI staining for cell cycle distribution. **(E)** Statistical analysis of cell cycle arrest in K562 cells. ∗∗*P* < 0.01 *vs.* vehicle control, *n* = 3.

Because inducing apoptosis is an efficient way to inhibit malignant proliferation of leukemic cells, then we examined whether compound DBH2 could induce the apoptosis and caused CML cell death. Flow cytometric results showed that compound DBH2 could induce the apoptosis of K562 cells, mainly induced leukemic cells going into early apoptotic stage, and the distinct populations of early apoptotic cells and late apoptotic/necrotic cells were increased significantly after treatment with compound DBH2 comparing to the vehicle control group (Fig. 1A, B). These data indicated that compound DBH2 could trigger significant apoptosis in CML cells with concentration-dependent responses. Furthermore, in comparison of control group, the typical apoptotic-body in nuclear appeared in K562 cells treated by 100 μg/mL compound DBH2 post 24 h (Fig. 1C). It has been reported that cell cycle arrest plays an important part in the anticancer effects, so we investigated the distribution of cells at different stages by flow cytometry. Following 100 μg/mL compound DBH2 treatment, the percentage of cells in the G2/M phase increased from 33.9% to 41.3%. Taken together, these findings demonstrated that compound DBH2 inhibited the viability of leukemic cells via inducing G2/M phase arrest and apoptosis in CML cells.

### Compound DBH2 induced apoptosis in bone marrow cell from SCL-tTA-BCR/ABL transgenic mice *in vitro*

**Figure 2:**
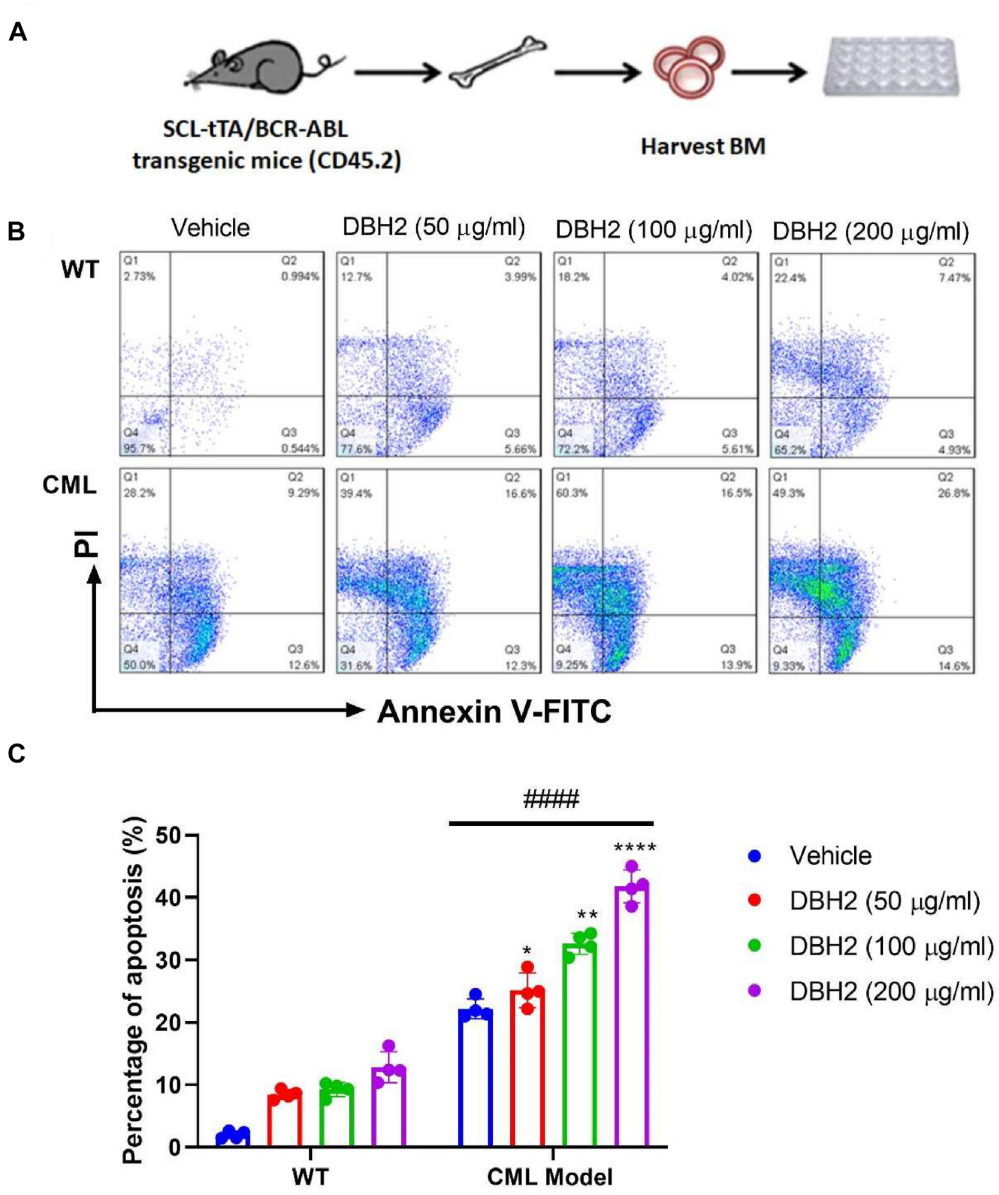
Compound DBH2 induced apoptosis of bone marrow cells from BCR-ABL gene transgenic mice. **(A)** The scheme of experimental procedure for bone marrow cells from SCL-tTA-BCR/ABL transgenic model mice. **(B)** The representative flow cytometry data of murine bone marrow cells after compound DBH2 treatment or 1% DMSO as vehicle control with Annexin-V and PI staining. **(C)** The quantitative analysis of apoptosis-based flow cytometry analysis. ∗*P* < 0.05, ∗∗*P* < 0.01, ∗∗∗∗*P* < 0.0001 *vs.* vehicle, ^####^*P* < 0.0001 *vs.* WT, *n* = 4.

Considering that human K562 cells were derived from a patient with CML, and the expression of both the normal Abl transcripts and the BCR-ABL fusion transcript decreased approximately ten-fold when the cells were induced to differentiate with hemin,^17^ we then established the inducible chronic phase of myeloid leukemia with expansion of hematopoietic stem cells in transgenic mice of BCR-ABL leukemogenesis. The induced apoptosis activity of compound DBH2 (50, 100, and 200 μg/mL) to the bone marrow cells from SCL-tTA-BCR/ABL transgenic model mice and wild type (WT) mice were detected. The illustration of bone marrow cells harvested from SCL-tTA-BCR/ABL transgenic model mice was present in Figure 2A, and subsequent detection of apoptosis in bone marrow cells was performed according to identical Annexin V-FITC/PI double staining. Consistently, compound DBH2 treatment could induce the remarkable apoptosis in bone marrow cells from CML model mice compared to the bone marrow cells from WT mice (Fig. 2B, C). These results further confirmed that compound DBH2 could lead much more significant apoptosis in pathological rather than in healthy hematopoietic progenitors.

### Suppression of CML development by compound DBH2 in combination with imatinib treatment *in vivo*

**Figure 3:**
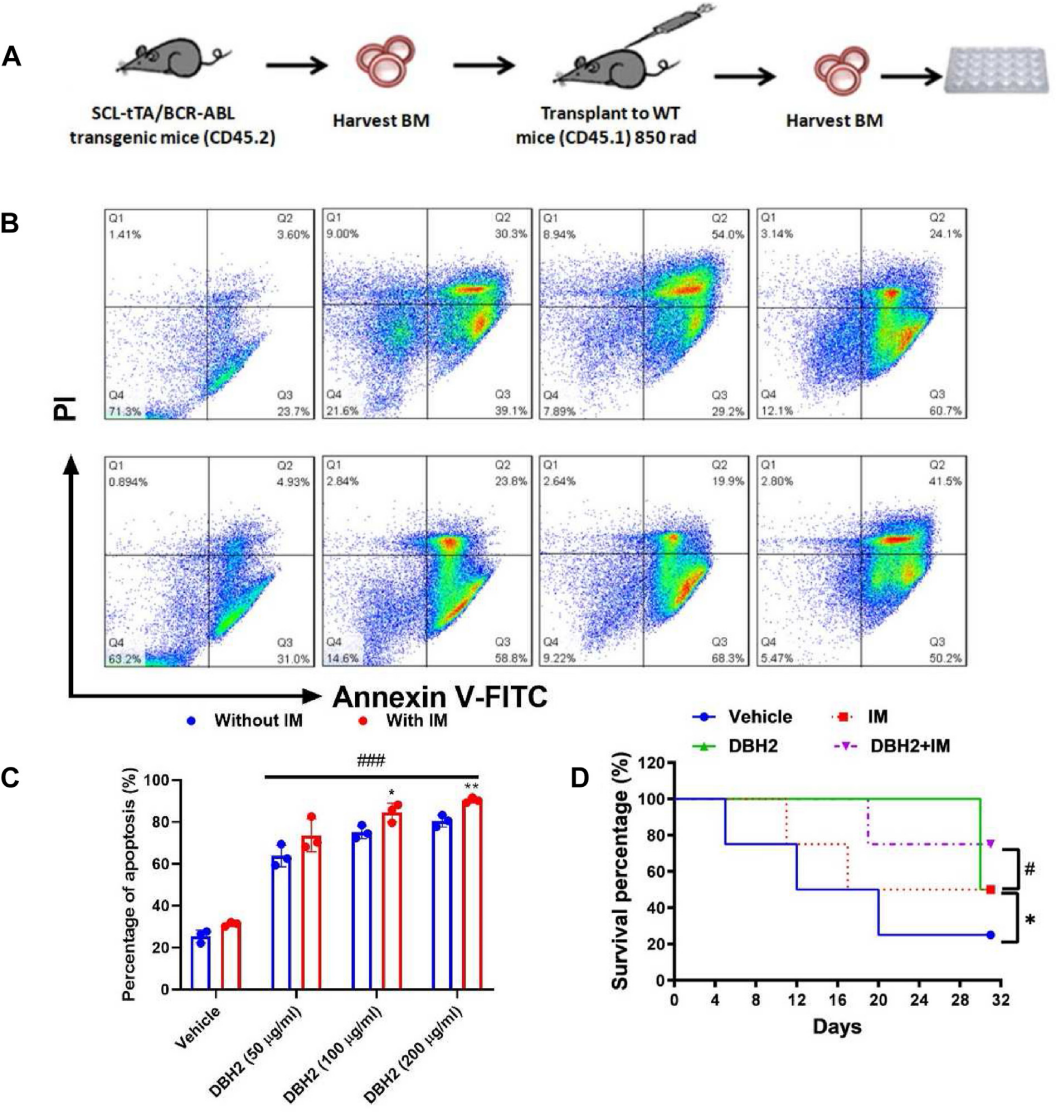
The effect of DBH2 in combination with imatinib (IM) on bone marrow cells from BCR-ABL gene transgenic mice *in vitro* and leukemic development *in vivo*. **(A)** Murine model was established and bone marrow transplantation was performed. After 2 months, bone marrow cells were collected and administrated with DBH2 (50, 100 and 200 μg/mL) and IM (1 μM) *in vitro*. **(B)** Flow cytometry analysis of murine bone marrow cells treated by DBH2 with or without IM. **(C)** The quantitative analysis of apoptosis-based flow cytometry analysis. ^###^*P* < 0.001 *vs.* vehicle control, ∗*P* < 0.05, ∗∗*P* < 0.01 *vs.* DBH2 alone treatment, *n* = 3. **(D)** Animal survival observation after intraperitoneal administration of 100 mg/kg DBH2, 50 mg/kg IM or DBH2 combined with IM (100 mg/kg; 50 mg/kg) for 32 days. ∗*P* < 0.05 *vs*. vehicle control, ^#^*P* < 0.05 *vs*. IM alone, *n* = 8.

Although TKIs such as imatinib played the definitive role in the treatment of CML based on their efficacy, the resistance was developed in some patients, and the simultaneous exposure of imatinib in combination with other drug minimized the risk and controls the leukemia. To explore whether compound DBH2 could be one of the potential partner with imatinib for CML treatment, using SCL-tTA-BCR/ABL transgenic model mice as donor and lethally irradiated wild type mice as receiver, bone marrow transplantation (BMT) was performed for establishing the BCR/ABL model mice in this study (Fig. 3A). After 8 weeks, the bone marrow reconstitution was confirmed, and the bone marrow cells of recipients were harvested and then treated by compound DBH2 at various concentrations with or without 1 μM imatinib. It was suggestive of an ascendant number of apoptotic cells induced by either compound DBH2 alone or in combination with imatinib (Fig. 3B). Comparing to compound DBH2 treatment alone, the combination of DBH2 (100 or 200 μg/mL) with imatinib could induce apoptosis significantly (Fig. 3C).

To evaluate the effect of compound DBH2 in combination with imatinib to the leukemic development *in vivo*, compound DBH2 (100 mg/kg), imatinib (50 mg/kg) or compound DBH2 in combination with imatinib (100 mg/kg; 50 mg/kg) were intragastric administrated to the recipients for 32 days with two-day interval post-BMT. Surprisingly, the survival of compound DBH2 alone treated mice was remarkably prolonged about two times, and comparing to the imatinib alone treatment, the survival of recipients was significantly prolonged 25% after compound DBH2 in combination with imatinib, and all the compound DBH2 in combination with imatinib treated mice were survival after 30-day experiment (Fig. 3D). Altogether, these data demonstrated that compound DBH2 could suppress the development of CML *in vivo*, and might be a promising candidate as a combination partner of imatinib for CML treatment.

### Compound DBH2 promoted apoptosis of bone marrow cells targeting leukemic stem cells and STAT pathway

**Figure 4:**
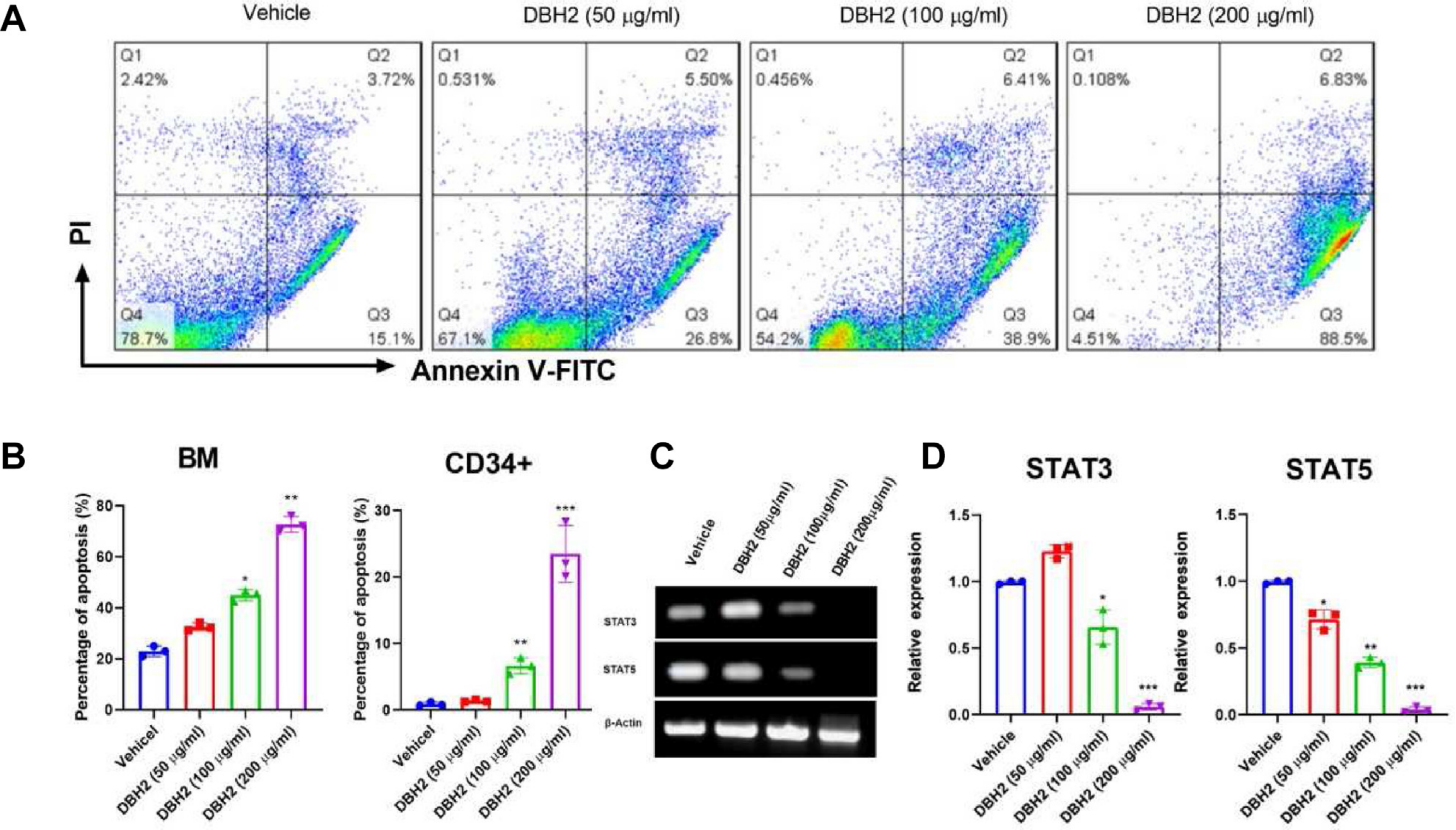
DBH2 promoted apoptosis targeting leukemic stem cells and STAT pathway. **(A)** Representative flow cytometric results of compound DBH2 (50, 100, and 200 μg/mL) and 1% DMSO treated bone marrow cells from CML patients. **(B)** The quantitative analysis of DBH2 induced bone marrow (BM) cells and CD34^+^ LSCs apoptosis from CML patients. ∗*P* < 0.05, ∗∗*P* < 0.01, ∗∗∗*P* < 0.001 *vs.* vehicle control, *n* = 3. **(C)** Expression of *STAT3* or *STAT5* mRNA in K562 cells after compound DBH2 (50, 100, and 200 μg/mL) treatment. **(D)** The quantitative analysis of relative expression of *STAT3* or *STAT5* mRNA after DBH2 (50, 100, and 200 μg/mL) and 1% DMSO treatment. ∗*P* < 0.05, ∗∗*P* < 0.01, ∗∗∗*P* < 0.001 *vs.* control, *n* = 3.

Because leukemic stem cells (LSCs) play an important role in the initiation and development of leukemia, and targeting of the LSCs holds considerable promise for more specific anti-leukemic therapies,^18^ we then set out to determine whether compound DBH2 could target the LSCs from CML patients. At first, similar to the previous results observed in K562 cells and bone marrow cells from BCR-ABL gene transgenic mice, compound DBH2 at 50, 100 and 200 μg/mL concentration promoted the apoptosis of bone marrow cells from CML patients in dose-dependent manner (Fig. 4A, B). Considering that CD34 was used as hematopoietic stem cells (HSC) markers, the CD34^+^ LSCs were isolated from the bone marrow cells of CML patients and treated with compound DBH2 for 24 h. We found that more than twice fold of CD34^+^ LSCs developed into the apoptotic process when treated by 100 μg/mL compound DBH2 (Fig. 4B). These data further demonstrated that compound DBH2 could promote the apoptosis of LSCs from CML patients.

Considering that the signal transducer and activator of transcription (STAT) family members play key role in many cellular processes including cell growth and apoptosis, we then observed the effect of compound DBH2 to the expression of STAT3 and STAT5 in the CML cells. The results indicated that compound DBH2 could inhibit the expression of *STAT3* and *STAT5* in a dose-dependent manner (Fig. 4C, D).

### Caspase-3 mediated the compound DBH2 induced apoptosis of CML cells

**Figure 5:**
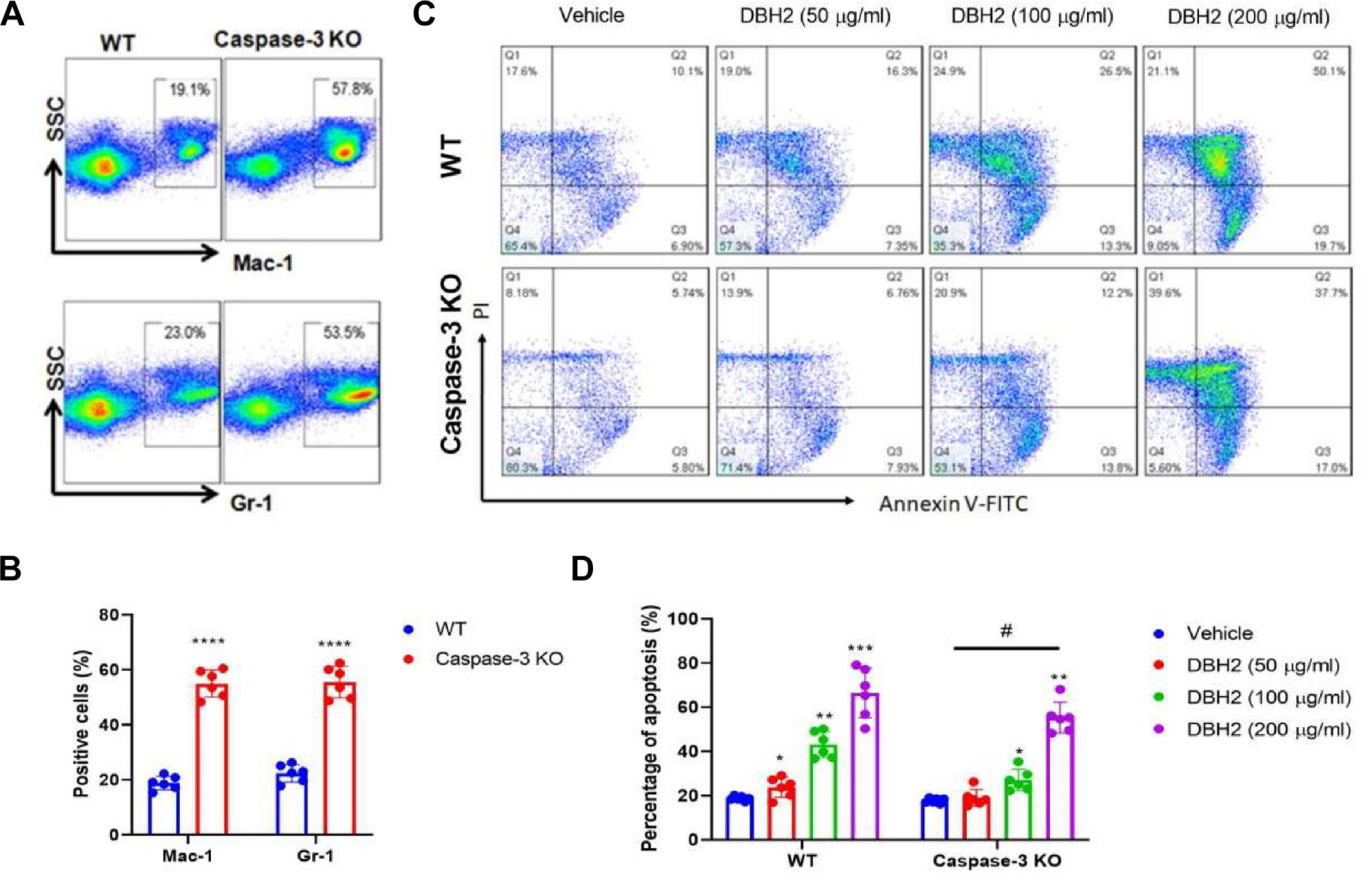
Caspase-3 mediated the apoptosis of leukemic cells induced by compound DBH2. **(A)** Representative flow cytometric results of Gr-1^+^/Mac-1^+^bone marrow myeloid cells from wild type (WT) and *caspase-3* gene knock-out mice. **(B)** The quantitative analysis of Gr-1^+^/Mac-1^+^bone marrow myeloid cells between WT and *caspase-3* knock-out groups. ∗∗∗∗*P* < 0.0001 *vs.* WT, *n* = 6. **(C)** Representative flow cytometric results of bone marrow myeloid cells from WT mice or *caspase-3* knock-out mice treated by compound DBH2 (50, 100, and 200 μg/mL) with 1% DMSO as vehicle control. **(D)** The quantitative analysis of apoptosis in bone marrow myeloid cells from WT mice or *caspase-3* knock-out mice after DBH2 (50, 100, and 200 μg/mL) treatment. ∗*P* < 0.05, ∗∗*P* < 0.01, ∗∗∗*P* < 0.001 *vs.* vehicle control, ^#^*P* < 0.05 *vs.* WT group, *n* = 6.

In molecular level, TKI could down-regulate the phosphorylation of STATs, induce caspase-3-dependent apoptosis, and the specific caspase-3 inhibitor could alleviate the apoptosis in human leukemia cells.^19^ Thus, we explore the role of caspase-3 in the anti-leukemia activity of compound DBH2 in *caspase-3* knock-out mice. Consistently, our results showed that Gr-1^+^/Mac-1^+^ immature myeloid cells were augmented in *caspase-3* knock-out mice (Fig. 5A, B), which confirmed that caspase-3 was involved in the myeloid progenitor abnormity. Next, the bone marrow cells were isolated from *caspase-3* gene knockout mice and subsequently administered by compound DBH2 (50, 100 and 200 μg/mL) for 24 h. Interestingly, flow cytometric analysis showed an increasing number of Annexin V positive cells in compound DBH2 treated bone marrow myeloid cells from both wild type mice and *caspase-3* gene knock-out mice, while compound DBH2-induced apoptosis was significant less in the bone marrow myeloid cells from *caspase-3* gene knock-out mice compared to the cells from wild type mice (Fig. 5C, D). Taken together, these results indicated that caspase-3 was involved in the compound DBH2-induced apoptosis in CML cells.

### Up-regulation of PARP1 and ROCK1 expression after compound DBH2 treatment in CML cells

**Figure 6:**
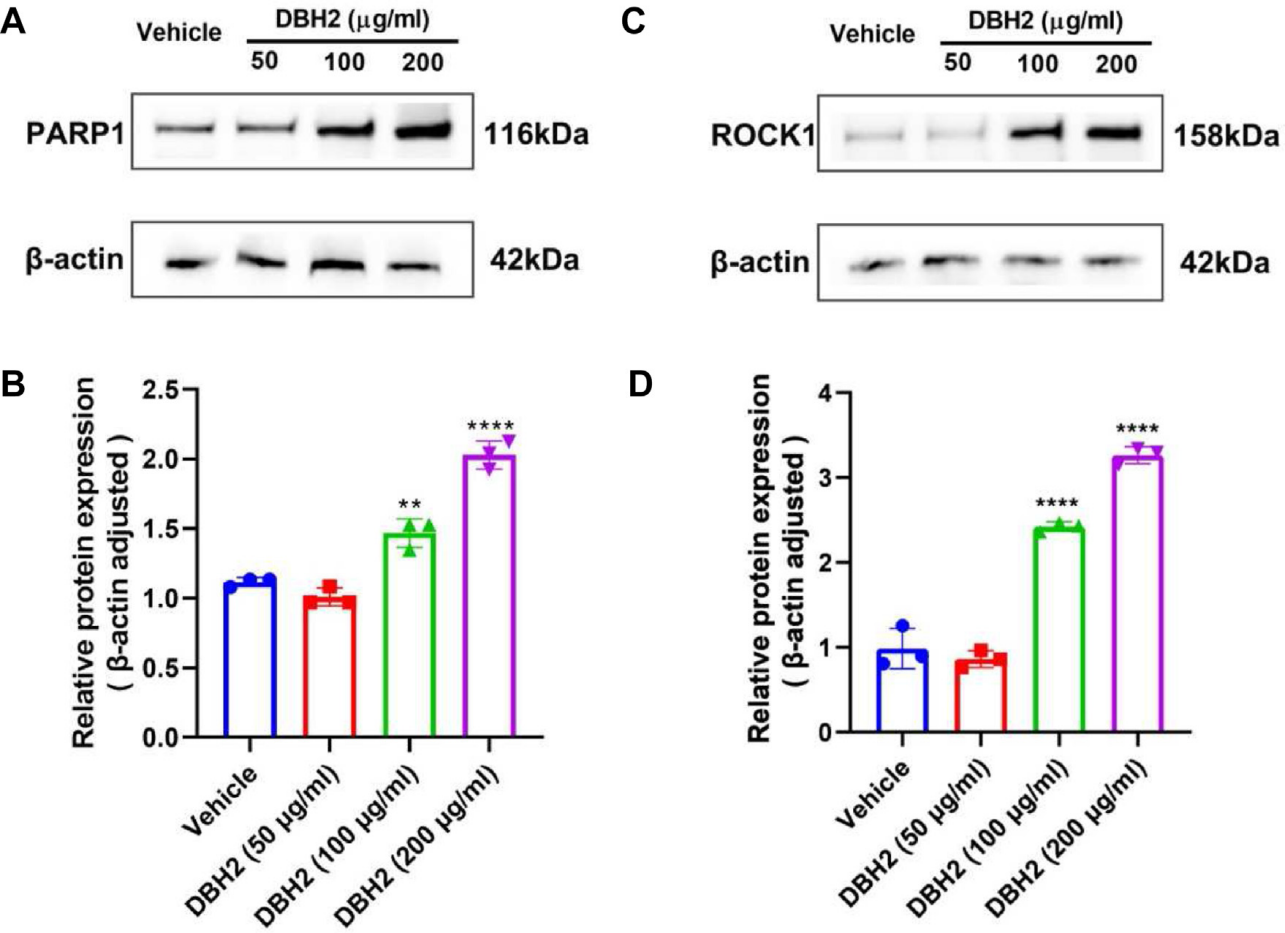
Compound DBH2 up-regulated the expression of PARP1 and ROCK1 in K562 cells. **(A)** The representative Western blot staining for PARP1 protein in the vehicle treatment control group and compound DBH2 (50, 100, and 200 μg/mL) treatment groups. **(B)** The quantitative analysis of PARP1 protein expression in the control and compound DBH2 treatment groups in K562 cells. ∗∗*P* < 0.05, ∗∗∗∗*P* < 0.0001 *vs*. vehicle control, *n* = 3. **(C)** The representative Western blot staining for ROCK1 protein in the vehicle treatment control group and compound DBH2 (50, 100, and 200 μg/mL) treatment groups. **(D)** The quantitative analysis of ROCK1 protein expression in the control and compound DBH2 treatment groups in K562 cells. ∗∗∗∗*P* < 0.0001 *vs*. vehicle control, *n* = 3.

Because caspase-3 is responsible for cleaving a number of cellular targets including the PARP, which plays the important role in both DNA repair and caspase dependent apoptosis.^20^ In order to observe the possible effect of compound DBH2 to the intracellular level of PARP1 in CML cells, the expression of PARP1 protein in K562 cells was measured after compound DBH2 treatment. Compared with the untreated control cells, Western blot assay showed that compound DBH2 at 100 and 200 μg/mL could up-regulate the expression of PARP1 significantly (Fig. 6A, B).

Meanwhile, caspase-3 also can cleave ROCK1 at a conserved DETD1113/G sequence, thereby removing the autoinhibitory C-terminal region and resulting in constitutively activation of ROCK1, and high-expression of ROCK1 modulated the apoptosis in many types of cells,^21^^,^^22^ thus we speculated that it might be involved in the compound DBH2 induced apoptosis of K562 cells. Our results showed that compound BDH2 up-regulated the expression of ROCK1 in dose-dependent manner (Fig. 6C, D). Consistently, these data indicated that compound DBH2 could promote the intracellular level of substrates of caspase-3 including PARP1 and ROCK1, thus resulting in the apoptosis of CML cells.

## Discussion

Coumarin derivatives have recently been reported to inhibit growth and induce apoptosis in malignancies. For example, in a number of multidrug resistant (MDR) cancer cells, 7-diethylamino-3(2′-benzoxazolyl)-coumarin could cause destabilization of microtubules, leading to a cell cycle arrest at G2/M stage.^23^ In human cervical cancer HeLa cells, coumarin also could induce morphological changes, and caused G0/G1 arrest and apoptosis with an IC_50_ of 54.2 mM.^24^ However, little is known about the activity of coumarin derivatives to the human CML. This study firstly confirmed the *in vitro* anti-leukemic activity of coumarin derivatives DBH2 in not only the K562 cells, but the bone marrow cells from SCL-tTA-BCR/ABL transgenic model mice and CML patients, and in contrast to the IC_50_ of coumarin derivatives reported in other cancer cells, these compounds inhibited the proliferation of CML cells much more effectively. Consistently, compound DBH2 also could induce the cell cycle arrest at G2/M stage.

As a result of the t(9; 22)(q34; q11.2), the chimeric BCR-ABL1 fusion protein was a constitutively active tyrosine kinase that leads to leukemogenesis, the introduction of tyrosine kinase inhibitor (TKI) treatment has resulted in dramatically improved survival in CML.^25^ With the development of a resistance to TKI in a significant proportion of leukemia patients, and the increasing understanding of CML biology, the strategy of TKI in combination with non-TKI agents were under investigation.^26^^,^^27^ In this study, we observed the efficacy of compound DBH2 to the TKI resistant K562 cells *in vitro*, and compound DBH2 in combination with imatinib in CL-tTA-BCR/ABL transgenic model mice *in vivo*, and the results showed the K562R cells were still sensitive to the compound DBH2, and imatinib in combination with compound DBH2 could significantly prolong survival rate and improve outcome. These data provided the promising strategy for CML treatment, however, more evidence such as pharmacokinetic and the best optimal concentration is to be explored for compound DBH2 as a potential candidate in this combination treatment strategy.

Although the BCR-ABL mutation is classically responsible for the apoptosis inhibition in CML, it was reported that irradiation could induce the DNA damage and apoptosis in many types of blood cells including lymphocytes and leucocytes. In mouse circulating lymphocyte, the percentage of apoptosis increased rapidly after 2, 4, 6 and 8 Gy of gamma-irradiation, and the apoptotic lymphocytes were 2.6, 3.8, 5.5, and 10.4 times higher than those in the controls, respectively.^28^ In human peripheral blood leukocytes, a significant increase in apoptosis was induced following gamma-irradiation with a dose dependent manner compared to that of controls.^29^ These evidences indicated the possible reason why the apoptosis was higher in CML mice model group than WT group in our system.

It is well known that the bone marrow microenvironment (niche) is responsible for the self-renew and differentiation of hematopoietic stem cells, while normal HSCs and LSCs interact with the stem cell niche bone marrow in different ways.^30^^,^^31^ Our present study revealed that compound DBH2 induced apoptosis in bone marrow cells from CML patients was almost twice fold compared to its effects on CD34^+^ LSC, indicating this small molecular might diminish the expansion of certain components of niche by activated apoptosis. Therefore, it was possible that compound DBH2 could disrupt niche/LSC interactions on long term durability of LSC proliferation in patients. This potential unique micro-environmental regulation of compound DBH2 will help us to understand its anti-leukemic mechanism. However, the precise cellular and molecular mechanism of compound DBH2 regulating LSC proliferation and niche maintenance is still required considerable further investigation.

During the past decades, intense research has focused upon the pathways that control caspase activation by STAT family, and increasing evidence showed that inhibitor of STAT3 and STAT5 suppressed proliferation and colony formation, and induced apoptosis by activation of caspase-3 cleavage in a number of cancer cells.^32^^,^^33^ In present study, compound DBH2 could inhibit the expression of STAT3 and STAT5 in dose-dependent manner, and it was confirmed that the myeloid lineage (Gr1^+^/Mac-1^+^) was augmented in *caspase-3* gene knock-out mice. Furthermore, compound DBH2 could also up-regulate the expression of PARP1 and ROCK1, both of which could be modulated in caspase-3 dependent apoptosis. These results indicated that STAT/caspase-3 signaling pathway might be involved in the mechanism of compound DBH2 induced apoptosis of CML cells.

In conclusion, we found that coumarin derivative decreased cell viability in both TKI sensitive and resistant CML cells *in vitro*, compound DBH2 in combination with imatinib suppressed leukemic development *in vivo,* and it induced apoptosis in CML cells depend on the activation of STAT3/STAT5/caspase-3 pathways. These findings highlight the therapeutic potential of coumarin derivative DBH2 for the treatment of drug-resistant CML.

## Ethics declaration

This project was reviewed and approved by the ethical committee of Tangdu Hospital, Air Force Medical University (No. 201903-87).

## Conflict of interests

The authors declare no conflict of interests.

## Funding

This project was supported by the 10.13039/501100004480Natural Science Foundation of Shaanxi Province, China (No. 2019JM-561).

## Appendix A Supplementary data

figs1 figs1
