# Supplementary figures and images for: A novel coumarin derivative DBH2 inhibits proliferation and induces apoptosis of chronic myeloid leukemia cells
Source: Genes Dis. 2022 Sep 8;10(2):596–607. doi: 10.1016/j.gendis.2022.08.021 (PMC10201669; doi:10.1016/j.gendis.2022.08.021)

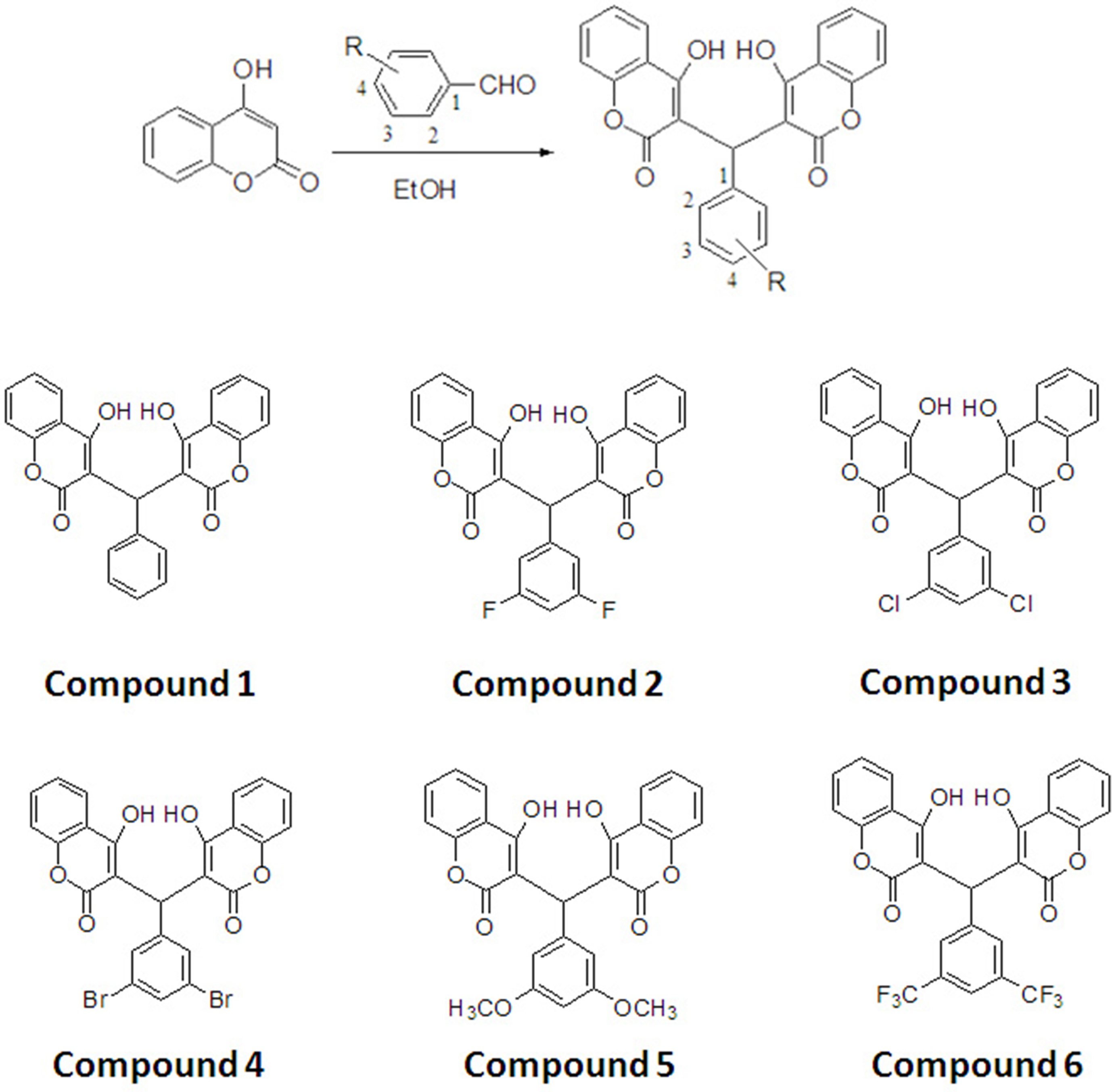

Supplement: figs1 [file figs1.jpg]
